# Projected Demographic Profile of People Living with HIV in Australia: Planning for an Older Generation

**DOI:** 10.1371/journal.pone.0038334

**Published:** 2012-08-09

**Authors:** James Jansson, David P. Wilson

**Affiliations:** The Kirby Institute, University of New South Wales, Sydney, Australia; Fudan University, China

## Abstract

**Background:**

Advances in HIV antiretroviral therapy (ART) has reduced mortality in people living with HIV (PLHIV), resulting in an ageing population of PLHIV. Knowledge of demographic details such as age, geographical location and sex, will aid in the planning of training and resource allocation to effectively care for the future complex health needs of PLHIV.

**Methods:**

An agent-based, stochastic, geographical model was developed to determine the current and future demographic of PLHIV in Australia. Data and parameters were sourced from Australia's National HIV Registry and peer reviewed literature. Processes that were simulated include progression to AIDS, mortality and internal migration.

**Findings:**

The model estimates the mean age of PLHIV in Australia is increasing at a rate of 0.49 years each year. The expected proportion of PLHIV in over 55 years is estimated to increase from 25.3% in 2010 to 44.2% in 2020. Median age is lower in inner-city areas of the capital cities than in rural areas. The areas with the highest prevalence of HIV will continue to be capital cities; however, other areas will have greater percentage growth from 2010 to 2020.

**Conclusions:**

The age of the population of people living with HIV is expected to increase considerably in the future. As the population of PLHIV ages, specialist clinical training and resource provision in the aged care sector will also need to be addressed.

## Introduction

Significant advances in HIV antiretroviral therapy (ART) has substantially decreased Acquired Immune Deficiency Syndrome (AIDS) associated mortality and morbidity [1], [2]. The increase in survival has meant that a growing proportion of people living with HIV (PLHIV) are elderly [3]. In Australia, the HIV epidemic remains highly concentrated among men who have sex with men (MSM), a well-educated and informed population with high access to publicly funded and freely available combination ART.

The nature of medical treatment required by PLHIV will change as they age. There is an increased risk of many health conditions associated with increased greater age; these include stroke and cardiovascular disease, cancers, frailty, kidney disease and liver disease [4]. However, there is strong evidence that HIV infection further increases the risk of many of these conditions [5]–[9]. The increasing numbers of PLHIV, and ageing nature of the population, means that there will be a greater number of people requiring complex clinical care including management of these chronic diseases along with HIV treatments with complicated antiretroviral regimens. The changing demographics of PLHIV require health systems to accommodate for the increased numbers of people with these complex healthcare requirements. Specialised and experienced clinicians are required to care for the needs of these patients but they are in relatively scarce supply [10]. Therefore, there is a need to plan for the health requirements of this important population, not only in magnitude but also according to demographic profiles, including specific geographical settings. By understanding the age structure of the population of PLHIV, necessary health care provisions can be made for those living with HIV ahead of time. Knowledge of the geographical distribution of people living with HIV can help us determine if the provisions made for PLHIV are appropriate for the area. Knowledge of both location and age of individuals allows for precise allocation of resources to maximise effective treatment and prevention on limited budgets.

HIV surveillance in Australia is based on routine case reporting of diagnoses of HIV being notified to local health authorities and then centrally collated at a national level. However, national registries do not monitor linkage into clinical care or other post HIV diagnosis events. Therefore, obtaining estimates of the profiles of populations of all PLHIV require mechanisms outside normal surveillance activities, such as the use of mathematical modelling.

While it can be helpful to discuss an epidemic in terms of averages and the largest affected population group, the finer elements of an epidemic need to be understood to fully grasp the nature of the epidemic and the most effective responses to it. In this study, we aim to project a detailed profile of the demographic characteristics of the population of PLHIV in Australia, based on Australia's National HIV Registry, migration patterns, geographical region-specific survival rates, and HIV/AIDS mortality ratios. These projections are carried out with the development of a detailed agent-based mathematical model. This approach has been applied in other settings, such as predictions of the HIV epidemic in Zimbabwe [11]. A previous model has produced estimations of the age of PLHIV in Australia who identify as MSM [12]. The current study advances previous work by utilising more sources of information, and incorporating greater demographic detail (such as geographical location and migration) to project several important characteristics about the future population of PLHIV in Australia.

## Methods

To better understand the current and future demographic and geographical profile of the population of PLHIV in Australia, an agent-based stochastic computer simulation model was developed. The model uses diagnosis case data from Australia's National HIV Registry to identify the initial locations of individuals, their age and sex. For each person, migration between areas was simulated as well as the probability of mortality. The model projected a 40 year time horizon and was run 1000 times. Diagnosis data were obtained from the National HIV Registry, up to the end of 2009. Relevant de-identified statistical information for each individual such as age, sex, postcode, and date of diagnosis were extracted from the registry for input into the model. Where data entries such as age or postcode were unavailable, these data were imputed by choosing, at random, values which are representative of the remaining data, including characteristics of associated year and state/territory of diagnosis; imputation was required for 0.7% of year of birth, 42% of postcode and 60% of CD4 count entries. Diagnoses after 2009 were assumed to occur at a similar rate and from similar demographics as in the previous five years (2005–2009). The demographic information of new future cases was sampled at random from existing records of those diagnosed from 2005 to 2009.

Migration of individuals was simulated to ensure that different death rates between Australian jurisdictions were taken into account and to determine the future location of PLHIV. Geographical stratification in the model occurred at the Statistical Local Area (SLA) level, as defined by 2006 Australian Standard Geographical Classification (AGSC) [13]. SLAs are the smallest reported region for the Australian census. Each individual was assigned a SLA at the date of diagnosis, according to their reported or imputed postcode in the National HIV Registry.

Migration rates between SLAs within jurisdictions and between jurisdictions were calculated from national census data collected by the Australian Bureau of Statistics (ABS), which measured the rates of movement from the years 2001 to 2006. The migration rates were used to form probabilities of a person moving from their existing location to another. Although simulation of migration occurred between statistical local areas, results were reported by AGSC Statistical Region [13] definitions, as they represented roughly similar numbers of people and were generally divided along geographically similar lines (inner city, suburban and regional). Reporting by Statistical Region also avoids the possibility of inadvertent identification of individuals in areas with low HIV prevalence.

The rate of death was based on age and time era. As such, development of AIDS was simulated prior to 1997. Initial CD4 counts were available for some individuals in the surveillance database, and where CD4 count was unavailable CD4 count was imputed from records of people with similar ages who did have a recorded CD4 level. Using the Kaplan-Meier curves on time until development of AIDS from Mellors et al. [14], a survival curve for each CD4 range was found of the form: 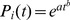, where P is the proportion of HIV-infected individuals who are not diagnosed with AIDS after time *t*, and *a* and *b* are constants that were determined by minimising the error between the Kaplan-Meier curve data from Mellors et al. [14] and the function Pi(t) above. The introduction of ART greatly reduced the rate of AIDS development, and hence simulation of incidence of AIDS was not considered to be consequential to the life expectancy of individuals beyond 1996. The mortality rate following the introduction of ART occurred in the context of the Australian guidelines for ART which recommend starting therapy when CD4<500/µL, strongly recommend commencing therapy at CD4<350/µL, and recommend treatment when CD4>500/µL only under certain circumstances. ART coverage in people living with diagnosed HIV is approximately 70–80% [15], [16], however ART coverage is not well monitored in the current Australian HIV surveillance system. ART is government subsidized but requires co-payments in some jurisdictions, which are considered to hinder ART uptake in 9.9% of PLHIV [16].

The mortality of individuals in the model was assumed to be a result of both HIV and non-HIV related causes. General population all-cause mortality rates were obtained from the ABS census. These data gave the mortality rates of the general population by year, age, sex, and state/territory of residence. Nakhaee et al. [17] produced estimates for standardised mortality ratios (SMR) of people living with HIV and living with AIDS in Australia for different time periods and ages. As a person in the simulation becomes infected and moves through their simulated life, at each year the appropriate mortality rate and SMR was selected based on the year, the person's age, sex, location and AIDS status. The mortality rate and SMR selected were then multiplied together to create a death probability for that year. A person's death was determined to occur by random selection based on the probability of death for that person's characteristics. For the purposes of determining mortality, transsexual individuals were considered to be male. Mortality parameters used in the model are presented in Table S3 and S4.

Uncertainty analysis was carried out to translate uncertainty in model inputs into output uncertainty ranges. Within simulations, the time until AIDS was sampled from a fixed distribution, estimated from Mellors et al [14]. A normal distribution was fit to the confidence intervals of the mortality risk ratios Nakhaee et al. [17]and Latin hypercube sampling was used to create a parameter space from which samples were drawn for each simulation. Uncertainty also existed in the number of new cases of HIV diagnoses expected in the future. The number of people diagnosed each year after 2009 was sampled from a normal distribution representative of the total numbers of infections in the years 2005 to 2009.

A total of 1000 simulations were performed over the years 1980 to 2020. Aggregate statistics (such as median and inter-quartile range) were determined for each of these simulations, and a mean was taken of these values to determine the expected outcome of all simulations.

## Results

**Figure 1:**
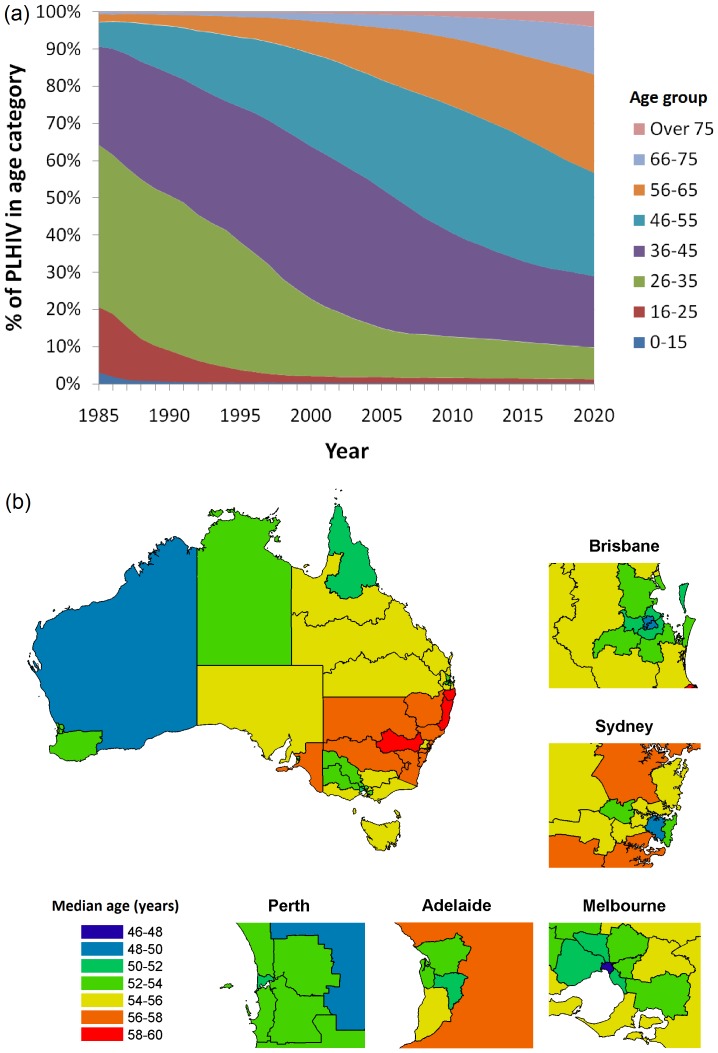
Estimated profile of people living with HIV in Australia (a) over time as an age distribution; (b) according to geographical distribution in the year 2020 based on average of the median age.

The estimated number of people living with diagnosed HIV infection in Australia in the past and projected to the year 2020 is presented in Table 1. According to our simulations, the population distribution of age of people living with HIV is rapidly shifting to older ages. The age structure of the population of PLHIV, shown in Figure 1a, has changed substantially since the early years of the epidemic. There was a substantially greater risk of mortality associated with HIV in the 1980s and early 1990s compared to the post-ART era. Despite the high mortality rate, the rate at which death occurred was insufficient to prevent the population from ageing substantially in this time period. The introduction of combination ART in the mid 1990s allowed for the proportion of people aged over 55 years to increase at an even greater rate. Our model estimates that the mean age of PLHIV in Australia is currently increasing by 0.49 years each year. The model suggests that the proportion of PLHIV aged 35 years or younger in 1990 was 50.7%, but dropped to just 12.6% by 2010, and will continue to decline to 9.9% by 2020. Conversely, the proportion of PLHIV aged over 55 years was estimated to be 11.1% in 2000, 25.3% in 2010 and substantially increase to 44.2% by 2020. Full results of population size by year and age group are given in Table S2. Although there has been a gradual increase in the median age at diagnosis (see Figure S1) and slight shift in the modes of transmission in Australia over recent years (see Figure S2), the distribution of age at diagnosis is not substantially different between population groups (see Figure S3) and thus epidemiological differences should not influence population age trends in the short-term future.

**Table 1:** The estimated number of people living with diagnosed HIV infection in Australia by year (median, 95% simulation range).

Year	Median Simulated Diagnoses	(95% simulation range)
1990	9575	(9483–9670)
1995	12630	(12526–12729)
2000	14136	(14016.5–14247)
2005	17235.5	(17121–17356)
2010	21404	(21257–21548.5)
2015	25646	(25413.5–25906.5)
2020	29661.5	(29363–29980.5)

The median age of people living with HIV varies by location, as shown in Figure 1b. Median age is least in inner-city areas of the capital cities, while rural areas are more likely to have greater median ages. Inner Sydney, NSW, for example, has a predicted median age of 49.3 years in 2020. Melbourne, Vic., has a predicted median age of 47.0 years, and Brisbane, QLD, has a predicted median age of 48.2 years. In comparison, statistical regions in the remainder of NSW have median ages of between 53.7 and 59.3 years and areas outside of inner Melbourne have median ages between 50.4 and 55.5 years.

**Figure 2:**
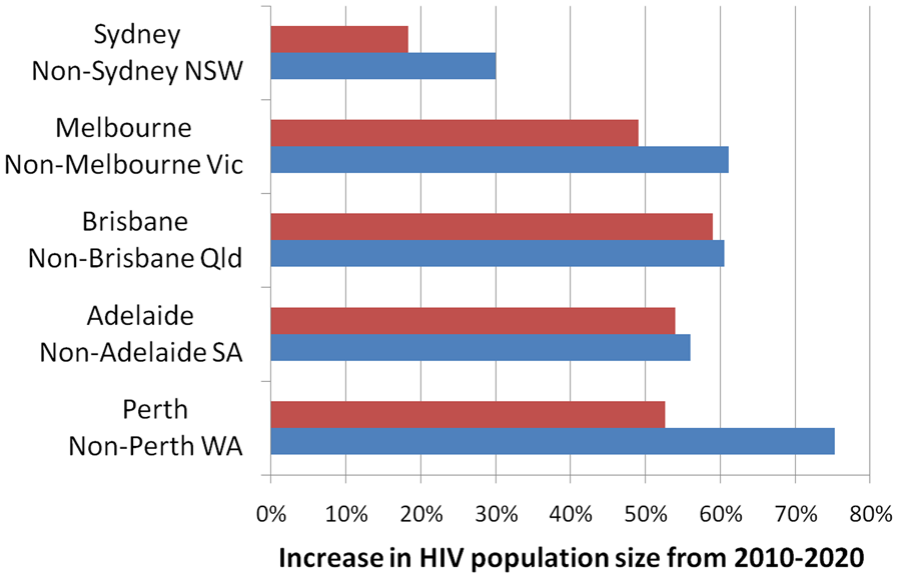
Comparison of metropolitan and non-metropolitan population change by jurisdiction. Only states that had separate statistical region definitions for metropolitan/non-metropolitan areas were included.

**Figure 3:**
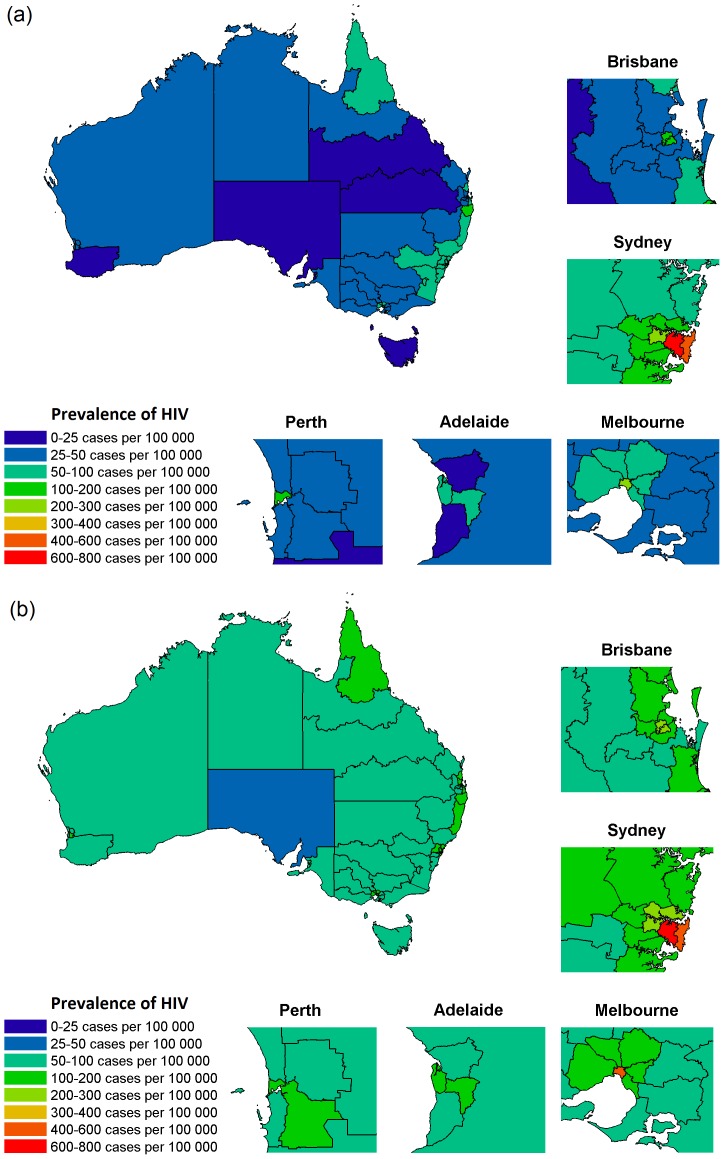
Prevalence of diagnosed HIV in Australia by statistical region in the year (a) 2000 and (b) 2020.

Migration is likely to become a major factor associated with the geography of Australia's HIV epidemic. The model predicts a difference in the relative change in the number of PLHIV between the major cities and the rural/regional areas of Australia for each jurisdiction (Figure 2). However, the simulations suggest that inner city areas of the major capital cities are where prevalence is likely to remain greatest. The geographical distribution of people living with diagnosed HIV in Australia in 2000 and projected distribution in 2020 are shown in Figure 3. The statistical region with the highest prevalence of PLHIV in all years is inner Sydney, with a population of 541 per 100,000 in 1990, 708 in 2010, and a projected prevalence of 700 per 100,000 in 2020. Other areas of high prevalence include inner Melbourne (414 per 100,000 in 2020), inner Brisbane (230 per 100,000 in 2020) and Sydney Eastern Suburbs (417 per 100000 in 2020); see Table S1 for projected population sizes of people living with diagnosed HIV across all statistical regions in Australia. General trends of net migration are suggestive of a pattern of people becoming diagnosed with HIV in high incidence/prevalence areas and then slowly moving away from those areas over time, in a fashion akin to spatial diffusion.

**Figure 4:**
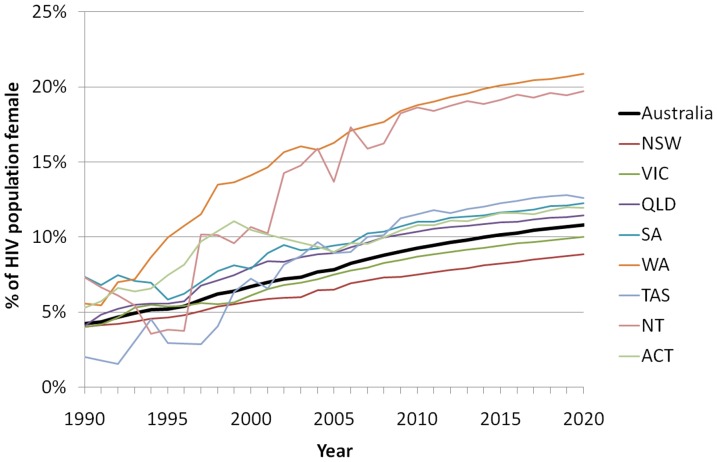
Proportion of PLHIV who are female by year and state/territory. The thick black curve represents all of Australia.

The sex composition of the population of PLHIV shows an increase in the number of females living with HIV. This is reflective of recent increases in diagnoses of heterosexually acquired HIV, particularly in non-Eastern jurisdictions [18] (Figure 4). In the Eastern jurisdictions (New South Wales, Queensland and Victoria), the epidemic is mainly concentrated in men (Figure 4). Although there are differences between jurisdictions, across all regions the sex profile is expected to remain predominantly male but with an increasing proportion of females. Nationally, 4.2% of PLHIV were estimated to be female in 1990 and this is expected to increase to 10.8% by 2020.

Uncertainty in the model outputs were determined by variations in rates of death, progression to AIDS and migration. We found that for the Australia wide prevalence in 2020, 95% of simulations were within +/−1.04% of the median of those simulations (median: 29661.5, 95% bound 29363–29981). On a statistical region basis, 95% of simulations on average fell within +/−10.8% of the median figure, with high prevalence statistical regions, such as inner Sydney, having relatively low uncertainty (+/−3.6%) and low prevalence statistical regions such as North-Western NSW having higher uncertainty (+/−26.31%). The results for age yielded little variation.

## Discussion

Our model has shown that the age of the population of people living with HIV has increased and is expected to increase considerably in the future. We found quantitative differences in average ages across Australian jurisdictions but some similarities qualitative trends of increasing ages. The increased age shown by this model is highly consistent with estimates by Murray et al [12]. The finding of increased age is common to populations of PLHIV across the world. It has been estimated that by 2015, over 50% of PLHIV in the USA will be aged over 50 years [19]. Our model projects similar age profiles in Australia (Figure 1a). In sub-Saharan Africa, an estimated 13% of HIV cases (or ∼3 million people) are aged 50 years or older [20]. HIV has become a complex chronic disease, that due to ageing, is characterised by increasing rates of numerous co-morbid conditions including liver and renal disease, cancers, bone disease, mental health disorders and cardiovascular diseases [21]. The increased rates of age-related disease not only puts increasing demands on healthcare systems, but also increases the risk of adverse reactions due to polypharmacy [22]. As the population of PLHIV ages substantially, issues such as HIV training and resource provision in the aged care sector will also need to be addressed, including access to effective diagnostics and treatments for these co-morbid disorders.

The model also demonstrates that HIV prevalence has been increasing and is expected to continue to increase. Increasing prevalence is an indication of the success of effective ART in reducing HIV/AIDS-related mortality. As the population size of PLHIV increases, appropriate health care provision will need to increase with it. However, the increase in service provision should not only occur by scaling up current clinical access points but to address geographical regions of emerging need. The migration of people within Australia, as simulated by this model, has done little to change the standing of the areas with the highest incidence also being the areas of high prevalence, namely in the major urban cities. But migration has lead to increased numbers of PLHIV in areas outside the traditional ‘hubs’ of HIV infection. Clinical services should be provided for all people living with HIV and although there is greatest demand in major cities, it is important to realise that there is a growing population of PLHIV who are not centrally located and require specialised services. The number of PLHIV in areas outside of the major cities is expected to increase at a greater rate than in the capital cities. The increase in service provision to those living outside of the major cities therefore needs to increase at a similar rate. Further, our model demonstrated that non-metropolitan areas have a greater median age than inner city areas. This age difference has implications for the healthcare sector, which is not as adequately resourced in areas outside major cities. In this study we have identified, for the first time, the specific locations that are likely to have the greatest needs.

Sex ratios in the epidemics also differ by region. Prevention strategies should be tailored to the demographics most at risk in each location, while still attempting to cover all at-risk groups. For example, in the Eastern States (of New South Wales, Victoria and Queensland) where the epidemics are largely concentrated among men, campaigns should focus on MSM, whereas in Western Australia where the epidemic affects more females, campaigns must also address heterosexual migrant workers and their sexual contacts.

Although great care has been taken in the parameterisation and construction of this model, like any model it has limitations. A limitation of this model is that the standardised mortality ratio of those living with HIV is sourced from a study that concluded in 2003 and mortality rates may have changed beyond 2003 and may continue to change as the demographic of PLHIV changes and as the effectiveness of ART continues to improve. As females exhibit a lower rate of mortality than males in the general population, part of the increase in female numbers is due to the increase in the survival rates of women. It is unknown whether the female standardised incidence ratio of HIV is higher or lower than for males, and hence this is a limitation with the model. There is also a limitation in the model with regard to the rates of movement of individuals between areas. The data that was used to inform the rate of movement was based on the ABS census, which measured movement of all Australians. The HIV epidemic has predominantly occurred in particular subgroups of the population such as MSM, relatively small groups of people who inject drugs and migrants from certain regions. These subgroups may make behavioural decisions which are different to the general population about where to live based on cultural and other factors. Inspection of the rates of movement from the ABS shows that the majority of movement occurs between SLAs that are relatively close to one another and hence this level of movement does not appear unreasonable if one assumes, for example, that some MSM would prefer not to live too far away from ‘gay scene’ areas.

An advantage of this model is that it is an agent-based model, using information of real individuals to inform the inputs and simulation of the model. While other studies have used agent-based modelling to simulate the transmission of HIV (e.g. [23], [24]) and the disease progression and mortality of people living with HIV [11], our model is somewhat unique in that it links to national surveillance case data and census data while also allowing for the modelling of disease progression and mortality by specific locations. This allows the model to capture the variability among PLHIV.

The qualitative conclusions of this Australian model may be generalised to other settings comparable to Australia, in particular, developed countries with relatively high levels of HIV care. Such countries can expect for their population of PLHIV to increasingly have a greater number of people in the older age groups in the coming years and that prevalence of infection may become more geographically spread. The ageing profile of populations of PLHIV has important implications for health systems, particularly with regards to co-morbid disorders, their treatment and interactions with complex HIV medications. This study can be used to assist in planning for addressing the clinical demands that are likely to emerge in the coming years, including provision of adequate specialised training and allocation of resources by specific regions to supply appropriate healthcare for PLHIV.

## Supporting Information

Figure S1 **Median age (in years) at HIV diagnosis by year.**
(PNG)

Figure S2 **Route of HIV exposure in people diagnosed by year of HIV diagnosis.**
(PNG)

Figure S3 **Median age at diagnosis (in years) over the period 2005–2009, by population category.**
(PNG)

Table S1 Model-based estimates of size of population of PLHIV by region.
(DOC)

Table S2 Model-based estimates of size of population of PLHIV by year and age group in years.
(DOC)

Table S3 Parameters used for HIV standardised mortality ratios (with 95% confidence interval) in Australia.
(DOC)

Table S4 Parameters used for AIDS standardised mortality ratios (with confidence interval) in Australia.
(DOC)
