# Supplementary figures and images for: Projected Demographic Profile of People Living with HIV in Australia: Planning for an Older Generation
Source: PLoS One. 2012 Aug 9;7(8):e38334. doi: 10.1371/journal.pone.0038334 (PMC3415409; doi:10.1371/journal.pone.0038334)

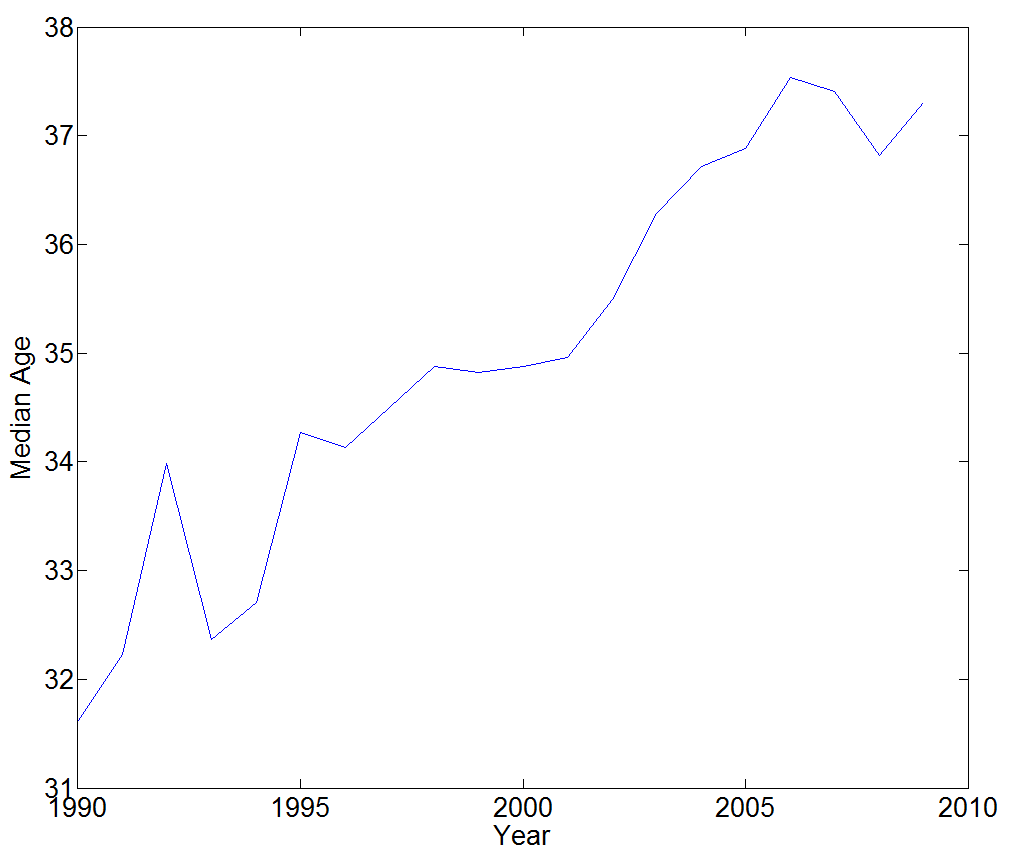

Supplement: Figure S1 — Median age (in years) at HIV diagnosis by year. (PNG) [file pone.0038334.s001.png]

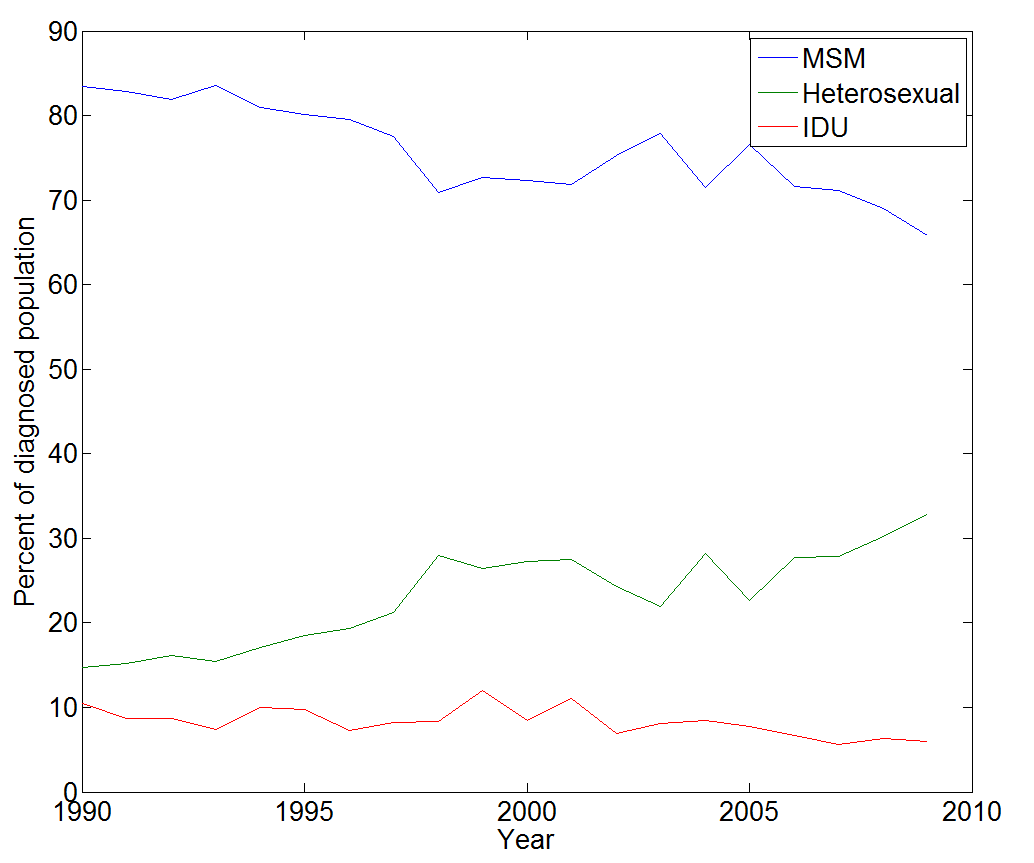

Supplement: Figure S2 — Route of HIV exposure in people diagnosed by year of HIV diagnosis. (PNG) [file pone.0038334.s002.png]

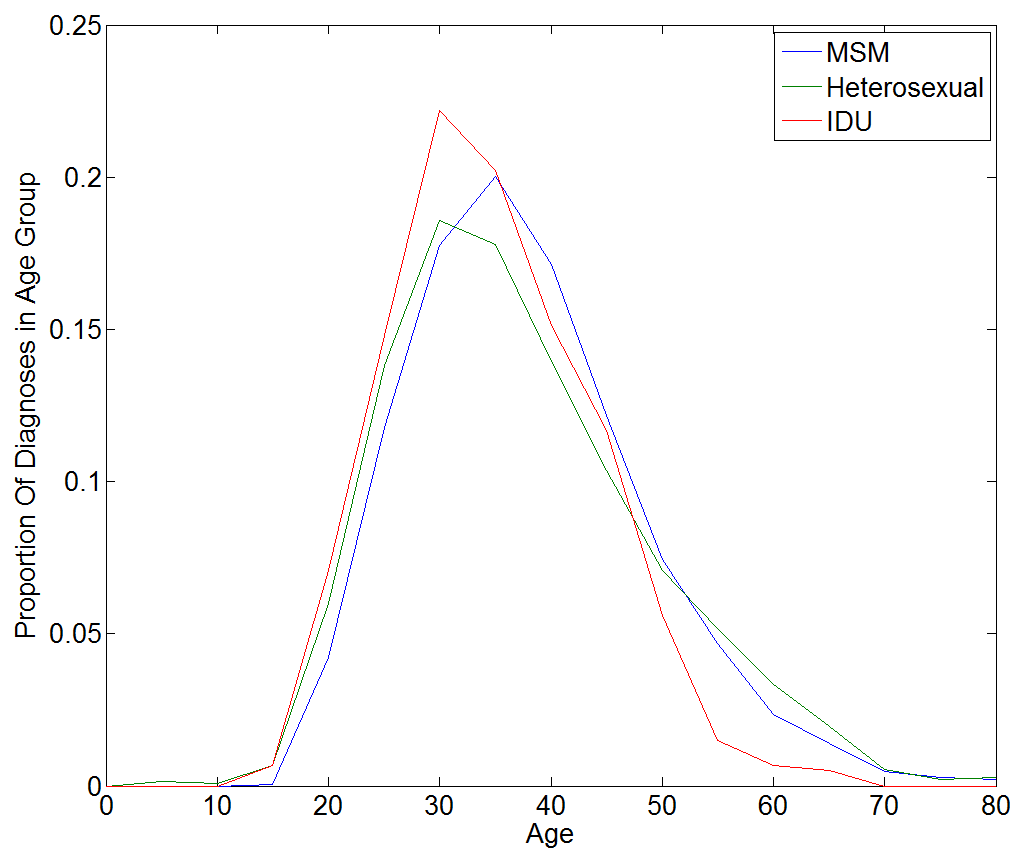

Supplement: Figure S3 — Median age at diagnosis (in years) over the period 2005–2009, by population category. (PNG) [file pone.0038334.s003.png]
