# Supplementary material for: Projected Demographic Profile of People Living with HIV in Australia: Planning for an Older Generation
Source: PLoS One. 2012 Aug 9;7(8):e38334. doi: 10.1371/journal.pone.0038334 (PMC3415409; doi:10.1371/journal.pone.0038334)
Supplement: Table S1 — Model-based estimates of size of population of PLHIV by region. (DOC) [file pone.0038334.s004.doc]

**Table S1: Model-based estimates of size of population of PLHIV by region**

|  |  | **Year** |  |  |
| --- | --- | --- | --- | --- |
|  | **1990** | **2000** | **2010** | **2020** |
| **NSW** | **6004** | **8053** | **10080** | **12168** |
| ***Sydney*** | **4865** | **6486** | **7995** | **9457** |
| Inner Sydney | 1395 | 1944 | 2342 | 2611 |
| Eastern Suburbs | 601 | 961 | 1053 | 1147 |
| St George-Sutherland | 444 | 508 | 616 | 745 |
| Canterbury-Bankstown | 268 | 320 | 429 | 529 |
| Fairfield-Liverpool | 162 | 273 | 331 | 378 |
| Outer South Western Sydney | 73 | 131 | 170 | 217 |
| Inner Western Sydney | 389 | 354 | 417 | 497 |
| Central Western Sydney | 386 | 373 | 432 | 498 |
| North Western Sydney | 454 | 475 | 574 | 715 |
| Lower Northern Sydney | 246 | 420 | 568 | 701 |
| Central Northern Sydney | 176 | 314 | 467 | 625 |
| Northern Beaches | 118 | 195 | 297 | 398 |
| Gosford-Wyong | 153 | 218 | 299 | 396 |
| ***Rural NSW*** | **1139** | **1567** | **2086** | **2711** |
| Hunter | 335 | 428 | 549 | 696 |
| Illawarra | 168 | 266 | 345 | 435 |
| South Eastern | 47 | 94 | 148 | 215 |
| Richmond-Tweed | 187 | 238 | 303 | 382 |
| Mid-North Coast | 186 | 218 | 282 | 362 |
| Northern | 33 | 64 | 105 | 146 |
| Far West-North Western | 28 | 46 | 65 | 89 |
| Central West | 99 | 120 | 140 | 168 |
| Murray-Murrumbidgee | 56 | 93 | 149 | 219 |
| **Victoria** | **1856** | **2685** | **4843** | **7304** |
| ***Melbourne*** | **1605** | **2299** | **4128** | **6152** |
| Outer Western Melbourne | 187 | 321 | 704 | 1147 |
| North Western Melbourne | 75 | 164 | 279 | 422 |
| Inner Melbourne | 537 | 636 | 1021 | 1338 |
| North Eastern Melbourne | 198 | 258 | 437 | 648 |
| Inner Eastern Melbourne | 258 | 328 | 535 | 781 |
| Southern Melbourne | 181 | 284 | 543 | 838 |
| Outer Eastern Melbourne | 89 | 130 | 207 | 313 |
| South Eastern Melbourne | 47 | 104 | 242 | 409 |
| Mornington Peninsula | 33 | 74 | 160 | 256 |
| ***Rural Victoria*** | **251** | **386** | **715** | **1152** |
| Barwon-Western District | 80 | 99 | 182 | 288 |
| Central Highlands-Wimmera | 37 | 59 | 97 | 159 |
| Loddon-Mallee | 37 | 62 | 151 | 254 |
| Goulburn-Ovens-Murray | 59 | 97 | 170 | 267 |
| All Gippsland | 38 | 69 | 115 | 184 |

|  |  | **Year** |  |  |
| --- | --- | --- | --- | --- |
|  | **1990** | **2000** | **2010** | **2020** |
| **Queensland** | **714** | **1766** | **3553** | **5675** |
| ***Brisbane*** | **383** | **870** | **1798** | **2858** |
| Brisbane City Inner Ring | 231 | 385 | 767 | 1121 |
| Brisbane City Outer Ring | 76 | 229 | 483 | 798 |
| South and East BSD Balance | 35 | 119 | 226 | 374 |
| North BSD Balance | 33 | 101 | 238 | 416 |
| Ipswich City | 8 | 36 | 84 | 149 |
| ***Rural Queensland*** | **331** | **896** | **1755** | **2817** |
| Gold Coast | 134 | 328 | 595 | 890 |
| Sunshine Coast | 58 | 133 | 232 | 366 |
| West Moreton | 5 | 18 | 37 | 67 |
| Wide Bay-Burnett | 19 | 67 | 148 | 258 |
| Darling Downs-South West | 13 | 47 | 115 | 195 |
| Mackay-Fitzroy-Central West | 20 | 74 | 165 | 287 |
| Northern-North West | 29 | 74 | 135 | 228 |
| Far North | 53 | 155 | 328 | 526 |
| **South Australia** | **344** | **535** | **963** | **1486** |
| ***Adelaide*** | **310** | **454** | **813** | **1252** |
| Northern Adelaide | 56 | 81 | 199 | 341 |
| Western Adelaide | 98 | 117 | 193 | 285 |
| Eastern Adelaide | 146 | 206 | 304 | 430 |
| Southern Adelaide | 10 | 50 | 117 | 196 |
| ***Rural South Australia*** | **34** | **81** | **150** | **234** |
| Northern and Western SA | 3 | 21 | 51 | 76 |
| Southern and Eastern SA | 31 | 60 | 99 | 158 |
| **Western Australia** | **448** | **742** | **1351** | **2116** |
| ***Perth*** | **415** | **633** | **1114** | **1701** |
| Central Metropolitan | 132 | 126 | 176 | 239 |
| East Metropolitan | 62 | 107 | 192 | 290 |
| North Metropolitan | 101 | 175 | 318 | 496 |
| South West Metropolitan | 49 | 97 | 179 | 281 |
| South East Metropolitan | 71 | 128 | 249 | 395 |
| ***Rural Western Australia*** | **33** | **109** | **237** | **416** |
| Lower Western WA | 18 | 45 | 104 | 191 |
| Remainder - Balance WA | 15 | 64 | 133 | 225 |
| **Tasmania** | **50** | **83** | **182** | **309** |
| **Northern Territory** | **41** | **76** | **145** | **218** |
| **Australian Capital Territory** | **113** | **191** | **269** | **369** |
