# Supplementary material for: Projected Demographic Profile of People Living with HIV in Australia: Planning for an Older Generation
Source: PLoS One. 2012 Aug 9;7(8):e38334. doi: 10.1371/journal.pone.0038334 (PMC3415409; doi:10.1371/journal.pone.0038334)
Supplement: Table S2 — Model-based estimates of size of population of PLHIV by year and age group in years. (DOC) [file pone.0038334.s005.doc]

**Table S2: Model-based estimates of size of population of PLHIV by year and age group in years**

| **Year** | **0-15** | **16-25** | **26-35** | **36-45** | **46-55** | **56-65** | **66-75** | **Over 75** |
| --- | --- | --- | --- | --- | --- | --- | --- | --- |
| **1985** | 75.5 | 418.8 | 1042.5 | 632.7 | 155.3 | 51.0 | 12.7 | 1.0 |
| **1986** | 81.7 | 697.3 | 1769.1 | 1181.6 | 300.2 | 78.3 | 27.2 | 2.9 |
| **1987** | 73.6 | 885.4 | 2675.7 | 1914.0 | 535.9 | 134.7 | 28.4 | 6.7 |
| **1988** | 68.0 | 856.9 | 3270.9 | 2413.1 | 782.4 | 183.1 | 42.5 | 7.7 |
| **1989** | 71.4 | 831.6 | 3708.4 | 2872.3 | 995.8 | 246.3 | 51.9 | 8.6 |
| **1990** | 62.8 | 799.8 | 3995.1 | 3143.1 | 1215.2 | 282.4 | 66.2 | 11.3 |
| **1991** | 61.8 | 755.3 | 4400.9 | 3549.1 | 1476.7 | 362.1 | 83.1 | 12.0 |
| **1992** | 58.8 | 669.6 | 4515.1 | 3949.9 | 1736.4 | 468.5 | 100.3 | 16.2 |
| **1993** | 56.5 | 587.6 | 4564.6 | 4167.9 | 1999.0 | 534.1 | 113.2 | 17.6 |
| **1994** | 60.5 | 507.4 | 4575.8 | 4309.1 | 2206.5 | 621.7 | 129.5 | 21.0 |
| **1995** | 55.6 | 424.3 | 4335.7 | 4591.9 | 2358.9 | 691.2 | 148.0 | 24.5 |
| **1996** | 57.5 | 360.8 | 4068.3 | 4807.1 | 2530.4 | 734.1 | 158.6 | 28.9 |
| **1997** | 62.3 | 292.7 | 3754.7 | 4961.9 | 2691.4 | 824.9 | 176.4 | 28.3 |
| **1998** | 57.3 | 270.9 | 3404.5 | 5324.8 | 2968.5 | 936.3 | 214.2 | 32.2 |
| **1999** | 58.8 | 243.5 | 3172.4 | 5572.6 | 3231.1 | 1068.9 | 257.7 | 36.7 |
| **2000** | 56.1 | 245.6 | 2930.9 | 5798.7 | 3521.0 | 1233.2 | 299.1 | 49.8 |
| **2001** | 57.3 | 249.3 | 2736.2 | 6005.5 | 3791.7 | 1383.6 | 353.4 | 58.6 |
| **2002** | 52.4 | 241.7 | 2639.8 | 6143.6 | 4082.6 | 1588.9 | 414.1 | 68.2 |
| **2003** | 49.4 | 256.7 | 2482.8 | 6293.9 | 4350.5 | 1848.9 | 485.7 | 80.5 |
| **2004** | 46.6 | 274.4 | 2374.9 | 6407.6 | 4665.8 | 2098.4 | 544.8 | 98.8 |
| **2005** | 47.7 | 286.3 | 2250.2 | 6460.4 | 5038.3 | 2405.4 | 627.3 | 118.3 |
| **2006** | 48.7 | 277.2 | 2214.7 | 6459.4 | 5488.1 | 2709.5 | 702.2 | 139.0 |
| **2007** | 56.7 | 273.0 | 2210.5 | 6388.2 | 5955.9 | 3010.7 | 811.1 | 162.3 |
| **2008** | 57.7 | 296.5 | 2265.7 | 6160.7 | 6466.8 | 3267.0 | 947.7 | 192.7 |
| **2009** | 60.5 | 295.5 | 2313.0 | 6091.4 | 6900.2 | 3557.0 | 1100.4 | 226.0 |
| **2010** | 62.7 | 308.0 | 2335.5 | 5971.6 | 7306.0 | 3893.5 | 1255.5 | 271.4 |
| **2011** | 63.0 | 312.2 | 2397.3 | 5845.3 | 7678.0 | 4210.9 | 1436.1 | 327.2 |
| **2012** | 65.8 | 307.8 | 2451.8 | 5806.3 | 7915.0 | 4544.6 | 1653.3 | 386.0 |
| **2013** | 69.1 | 305.0 | 2493.5 | 5690.7 | 8195.3 | 4888.5 | 1894.0 | 445.6 |
| **2014** | 69.8 | 309.6 | 2513.2 | 5642.7 | 8412.6 | 5214.1 | 2149.7 | 511.9 |
| **2015** | 69.8 | 314.7 | 2509.2 | 5567.5 | 8543.2 | 5628.5 | 2430.0 | 590.9 |
| **2016** | 74.3 | 312.1 | 2517.4 | 5545.7 | 8592.8 | 6064.9 | 2701.3 | 667.9 |
| **2017** | 77.6 | 312.4 | 2523.9 | 5542.6 | 8559.9 | 6528.0 | 2979.4 | 766.2 |
| **2018** | 76.4 | 315.8 | 2522.0 | 5632.2 | 8382.1 | 7041.6 | 3230.3 | 894.2 |
| **2019** | 78.7 | 316.9 | 2517.7 | 5660.4 | 8337.2 | 7440.0 | 3496.6 | 1034.8 |
| **2020** | 77.5 | 320.1 | 2528.4 | 5683.9 | 8240.0 | 7832.6 | 3798.5 | 1179.6 |
