# Supplementary material for: Projected Demographic Profile of People Living with HIV in Australia: Planning for an Older Generation
Source: PLoS One. 2012 Aug 9;7(8):e38334. doi: 10.1371/journal.pone.0038334 (PMC3415409; doi:10.1371/journal.pone.0038334)
Supplement: Table S3 — Parameters used for HIV standardised mortality ratios (with 95% confidence interval) in Australia. (DOC) [file pone.0038334.s006.doc]

**Table S3: Parameters used for HIV standardised mortality ratios (with 95% confidence interval) in Australia .**

| **Age  (years)** | **Year** | | |
| --- | --- | --- | --- |
|  | **1980-1989** | **1990-1996** | **1997-2020** |
| **0-24** | 4.89 (2.9-8.26) | 2.76 (1.92-3.98) | 3.79 (2.39-6.01) |
| **25-34** | 4.21 (3.13-5.65) | 1.89 (1.63-2.19) | 2.69 (2.35-3.07) |
| **35-44** | 4.04 (2.97-5.51) | 1.52 (1.63-2.19) | 2.16 (1.96-2.38) |
| **45-54** | 1.15 (0.58-2.32) | 1.08 (0.89-1.31) | 1.56 (1.39-1.76) |
| **55-64** | 1.36 (0.65-2.86) | 0.46 (0.33-0.65) | 1.05 (0.89-1.24) |
| **65+** | 0.83 (0.27-2.58) | 0.49 (0.34-0.72) | 0.68 (0.54-0.84) |

**References**

1. Nakhaee, F., et al., *Changes in mortality following HIV and AIDS and estimation of the number of people living with diagnosed HIV/AIDS in Australia, 1981–2003.* Sexual Health, 2009. **6**(2): p. 129-134.
