# Supplementary material for: Projected Demographic Profile of People Living with HIV in Australia: Planning for an Older Generation
Source: PLoS One. 2012 Aug 9;7(8):e38334. doi: 10.1371/journal.pone.0038334 (PMC3415409; doi:10.1371/journal.pone.0038334)
Supplement: Table S4 — Parameters used for AIDS standardised mortality ratios (with confidence interval) in Australia. (DOC) [file pone.0038334.s007.doc]

**Table S4: Parameters used for AIDS standardised mortality ratios (with confidence interval) in Australia .**

| **Age**  **(years)** | **Year** | | |
| --- | --- | --- | --- |
|  | **1980-1989** | **1990-1996** | **1997-2020** |
| **0-24** | 310.45 (219.54-439) | 78.18 (61.64-99.15) | 19.95 (11.05-36.02) |
| **25-34** | 256.49 (229.47-286.69) | 59.9 (56.57-63.43) | 14.11 (12.3-16.19) |
| **35-44** | 209.58 (190.74-230.28) | 46.01 (43.88-48.24) | 8.12 (7.43-8.87) |
| **45-54** | 95.64 (83.29-109.81) | 41.13 (23.46-26.52) | 4.25 (3.81-4.472) |
| **55-64** | 39.72 (30.42-51.86) | 9.25 (8.24-10.32) | 2.43 (2.08-2.84) |
| **65+** | 37.81 (26.88-53.18) | 4.44 (3.6-5.47) | 1.19 (0.94-1.5) |

**References**

1. Nakhaee, F., et al., *Changes in mortality following HIV and AIDS and estimation of the number of people living with diagnosed HIV/AIDS in Australia, 1981–2003.* Sexual Health, 2009. **6**(2): p. 129-134.
